# Enhancing endorsement of scientific inquiry increases support for pro-environment policies

**DOI:** 10.1098/rsos.160360

**Published:** 2016-09-28

**Authors:** Aaron Drummond, Matthew A. Palmer, James D. Sauer

**Affiliations:** 1 School of Education, Flinders University, Adelaide, South Australia 5001, Australia; 2 School of Psychology, Massey University, Palmerston North 4442, New Zealand; 3 Department of Psychology, School of Medicine, University of Tasmania, Launceston, Tasmania 7250, Australia; 4 Department of Psychology, School of Medicine, University of Tasmania, Hobart, Tasmania 7001, Australia

**Keywords:** climate change, policy making, science communication, scientific literacy

## Abstract

Pro-environment policies require public support and engagement, but in countries such as the USA, public support for pro-environment policies remains low. Increasing public scientific literacy is unlikely to solve this, because increased scientific literacy does not guarantee increased acceptance of critical environmental issues (e.g. that climate change is occurring). We distinguish between scientific literacy (basic scientific knowledge) and endorsement of scientific inquiry (perceiving science as a valuable way of accumulating knowledge), and examine the relationship between people's endorsement of scientific inquiry and their support for pro-environment policy. Analysis of a large, publicly available dataset shows that support for pro-environment policies is more strongly related to endorsement of scientific inquiry than to scientific literacy among adolescents. An experiment demonstrates that a brief intervention can increase support for pro-environment policies via increased endorsement of scientific inquiry among adults. Public education about the merits of scientific inquiry may facilitate increased support for pro-environment policies.

Science is much more than a body of knowledge. It is a way of thinking. This is central to its success … We need wide appreciation of this kind of thinking. It works. It's an essential tool for a democracy in an age of change.

Carl Sagan, 1990, paragraph 9 [1]

No challenge poses a greater threat to future generations than climate change.

Barack Obama, 2015 [2]

A quarter of a century on, Sagan's quote strikes at the core of contemporary debate on climate change. Pro-environment policies to counteract the effects of climate change require public support and engagement [3,4]. However, in countries such as the USA, public support for pro-environment policies is well below consensus levels [5]. Basic scientific literacy is defined as a level of scientific competence sufficient to comprehend public debate about science and technology in the media [6]. Although scientific literacy is a complex construct, Miller, Durant and colleagues—who developed measures of scientific literacy used in large-scale surveys including the Eurobarometer and US National Science Foundation's (NSF) Science and Engineering Indicators program [7]—conceptualized two key components [6,8,9]. The first involves knowledge of facts and concepts relevant to core areas of science. Examples from the NSF indicators include a basic knowledge of the structure of molecules (an electron is smaller than an atom), the nature of the universe (the Earth orbits the Sun) and genetics (the father's gene determines a child's sex). The second involves understanding the process of scientific inquiry, encompassing a basic understanding of concepts including probability, the use of empirical evidence to evaluate propositions and the use of experimentation to develop theories and test hypotheses.

Intuitively, poor scientific literacy may undermine public understanding of complex environmental issues for which there is strong scientific support. For example, given the overwhelming consensus among scientists that climate change is occurring [10,11], researchers have acknowledged that it might be tempting to think that lower acceptance of the evidence for climate change among the public might be attributable to poor scientific literacy [12–14]. If so, enhancing scientific literacy should increase public acceptance that climate change is occurring and public support for pro-environment policies (assuming, of course, the publics’ motivation and ability to process the evidence analytically [15]). However, greater scientific literacy does not entail greater acceptance of evidence for climate change. When examined across a large sample with diverse socio-cultural views, the relationship between scientific literacy and climate change beliefs varies depending on respondents’ dominant world views [16]. Among people with egalitarian, communitarian world views, scientific literacy is positively associated with belief that climate change is occurring. However, among people with hierarchical, individualistic world views, scientific literacy is negatively associated with belief that climate change is occurring. Consequently, beliefs about climate change are most polarized (along socio-political lines) among the most scientifically literate members of the public [16]. Although these findings do not address the relationship between acceptance of climate change and scientific knowledge specifically pertaining to climate change [17,18], they demonstrate that increasing the public's basic scientific literacy is unlikely to resolve debate about climate change or increase support for pro-environment climate policy, and may actually exacerbate disagreement between those holding polarized socio-political world views.

We investigated the relationship between scientific literacy and support for pro-environment policy from a different viewpoint. A critical factor affecting support for pro-environment policies might be the attitude that people hold about the role of scientific research in acquiring knowledge and developing policy. We term this concept *endorsement of scientific inquiry*, defined as the extent to which a person (i) endorses the process of scientific inquiry as a valuable way of accumulating knowledge and (ii) endorses the results of relevant scientific research as a valid basis for making decisions and taking action to achieve societal goals. This construct overlaps to some extent with several other constructs that have been investigated in relation to scientific literacy [9,19] but it is nevertheless distinct from them. For example, knowledge of scientific facts and understanding of the scientific method are important aspects of scientific literacy [8,9], but they are not necessary for endorsement of scientific inquiry; someone with little knowledge of scientific facts can still accept that scientific research is the best way to develop knowledge in a range of areas. Endorsement of scientific inquiry is also distinct from interest in science—either in terms of one's own participation in scientific research or in learning about new scientific discoveries [8,9]—because having an interest in science does not necessarily imply that one believes scientific research should guide decision-making or policy development. Conversely, believing that scientific research should guide policy does not necessarily imply that one has a personal interest in science.

Perhaps the construct that is most closely aligned with endorsement of scientific inquiry is attitude regarding the overall impact of science on society [7,9]. This has been measured, for example, by asking questions about benefits of science for society (e.g. whether science makes the world better or worse off; whether science makes lives healthier, easier and more comfortable) and reservations about science (e.g. whether science makes our way of life change too fast). We would expect such attitudes to covary to some extent with endorsement of scientific inquiry. In particular, the degree to which one thinks science improves the world should be linked to one's attitudes towards basing decisions on scientific findings (i.e. if a person thinks science improves our quality of life, he or she should be inclined to support basing decision-making and policy on scientific findings). However, these two constructs are distinct because attitudes towards the impact of science on society do not capture views about science as a valid means of accumulating knowledge, which we propose is an important component of endorsement of scientific inquiry.

We also distinguish between endorsement of scientific inquiry and support for government funding of science [9]. Although we would expect these constructs to co-vary in many situations, there are circumstances under which they will diverge. For example, variations in economic conditions might affect attitudes towards spending government money on scientific research, but should have no effect on endorsement of scientific inquiry as a means of accumulating knowledge. Moreover, it is possible to simultaneously endorse scientific inquiry while believing that greater funding for science should come from industry partnerships and non-governmental sources. Finally, we note that endorsement of scientific inquiry differs from the ethical endorsement of scientific research in specific, controversial domains [20]. For example, one can agree that science enables the accumulation of valid knowledge, without supporting the use of scientific research to advance the development of nuclear weapons or human cloning.

Why might support for pro-environmental policies be better predicted by endorsement of scientific inquiry than scientific literacy? The answer may reflect reliance on Type 1 (cf. Type 2) decision-making. We use the terminology ‘Type 1’ and ‘Type 2’ to encompass various instantiations of dual process theory that include two sets of decision-making systems: one being essentially automatic and intuitive (Type 1), the other essentially deliberative and analytical (Type 2) [21–23]. Scientific literacy indexes knowledge of scientific facts, not acceptance of those facts. For example, while knowledge of the theory of evolution is widespread, there remains considerable public disagreement over the accuracy of the theory [24]. When considering a contentious issue, even scientifically literate audiences will typically not critique the relevant scientific evidence themselves (i.e. by reading research articles in academic journals). Thus, their acceptance of the scientific evidence is likely to reflect Type 1 message factors (source credibility, compatibility with existing beliefs, etc.). Accordingly, for many issues, individuals' interpretation of facts and, subsequently, their application of accumulated scientific knowledge to gain understanding can be coloured by their broader socio-political belief system [25]. Thus, acceptance of a scientifically supported position may depend on the extent to which it conforms to available heuristics. Endorsement of scientific inquiry, by contrast, indexes belief in the importance of scientific inquiry *as a means to understanding*. Thus, in the context of pro-environment policy, individuals' acceptance of scientific consensus on environmental issues might hinge on whether they view scientific research as a valid method of accumulating knowledge, rather than their accumulated scientific knowledge. The former construct relates directly to the perception of scientific inquiry as a credible source of information. The latter does not. Put another way, knowing the theoretical underpinnings of bloodletting does not entail acceptance of its therapeutic efficacy.

If our reasoning is correct, endorsement of scientific inquiry will be a stronger predictor than scientific literacy of support for pro-environment policies. Moreover, if endorsement of scientific literacy can be increased, this may lead to greater support for pro-environment policies. Two studies tested these ideas. In Study 1, we analysed data from the publically available 2006 Programme of International Student Assessment (PISA) [26] survey of students to examine the extent to which support for environmental policies was predicted by endorsement of scientific inquiry and scientific literacy. In Study 2, we conducted an experiment to test whether a brief intervention designed to increase endorsement of scientific inquiry would, in turn, increase support for pro-environmental policies.

## 1. Study 1

In Study 1, we analysed publically available data from the 2006 PISA survey that included measures of scientific literacy, endorsement of scientific inquiry and endorsement of pro-environment policies [26–28]. We expected this cross-sectional study to demonstrate that endorsement of scientific inquiry was more strongly predictive than scientific literacy of support for pro-environment policies.

### 1.1. Method

#### 1.1.1. Dataset

The Programme for International Student Assessment (PISA), run by the Organization for Economic Co-operation and Development (OECD), examines the relative performance of students (aged approx. 15 years) in science, mathematics and literacy. The 2006 survey included data from 398 750 students across 58 countries. In addition to standardized tests of scientific literacy, the 2006 PISA tests included items assessing attitudes towards science and support for a range of environmental policies. We reanalysed data from these scales.

##### 1.1.1.1. Scientific literacy

Scientific literacy was measured via a series of multiple choice (e.g. What is the role of bacteria in dental caries?) and short answer items (e.g. Give one reason why there is less bacteria and particle pollution in ground water than in water from surface sources such as lakes and rivers). Note that PISA has only released examples of the items used to test scientific literacy [28]. The scientific literacy scale is calculated by PISA, has been extensively validated [29] and has a Cronbach's *α* of 0.86, representing excellent internal reliability.

##### 1.1.1.2. Endorsement of scientific inquiry

The *endorsement for scientific inquiry* scale (labelled *Support for Scientific Inquiry* in the PISA dataset) assessed students' perceptions of the importance of considering scientific perspectives, support for the use of facts and rational explanations, and the need for logical and careful processes when drawing conclusions. Endorsement of scientific inquiry was measured via students' level of agreement with a series of statements (see table 1 for a sample, provided by PISA, of the items used). Students rated their agreement on a scale from 1—*strongly agree* to 4—*strongly disagree*. The endorsement scale is calculated and validated by PISA [29], and has high reliability, *α* = 0.73.

**Table 1.** Items in the endorsement of scientific inquiry and pro-environment policy support scales.

endorsement of scientific inquiry sample items
(a) Preservation of ancient ruins should be based on scientific evidence concerning the causes of damage.
(b) Statements about the causes of acid rain should be based on scientific research.
(c) The systematic study of fossils is important.
(d) Scientific investigation of geological layers is important.
(e) Action to protect national parks from damage should be based on scientific evidence.
(f) I am in favour of research to develop vaccines for new strains of influenza.
(g) The cause of a disease can only be identified by scientific research.
(h) The effectiveness of unconventional treatments for diseases should be subject to scientific investigation.

support for pro-environment policy items
(a) It is important to carry out regular checks on the emissions from cars as a condition of their use.
(b) It disturbs me when energy is wasted through the unnecessary use of electrical appliances.
(c) I am in favour of having laws that regulate factory emissions even if this would increase the price of products.
(d) To reduce waste, the use of plastic packaging should be kept to a minimum.
(e) Industries should be required to prove that they safely dispose of dangerous waste materials.
(f) I am in favour of having laws that protect the habitats of endangered species.
(g) Electricity should be produced from renewable sources as much as possible, even if this increases the cost.

##### 1.1.1.3. Support for pro-environment policies

Support for pro-environment policies (labelled *environmental responsibility* in the PISA data) was measured via level of agreement with seven statements (table 1) with ratings made on a scale from 1—*strongly agree* to 4—*strongly disagree* [28–30]. The support for pro-environment policies scale has high internal consistency, *α* = 0.73, and has been validated against similar constructs by PISA [29].

For the scientific inquiry and pro-environmental policy scales, items were reverse scored such that higher scores represented greater endorsement of scientific inquiry and support for pro-environment policies. *Scientific literacy* and *endorsement of scientific inquiry* are supplied by PISA on a scale with an international average of approximately 500 and a standard deviation of approximately 100. To ensure consistency, we also transformed *support for pro-environment policy* scores to a scale with an international average of approximately 500 and a standard deviation of approximately 100.

### 1.2. Results

We analysed the data using a multilevel, linear mixed modelling approach with endorsement of pro-environment policies as the outcome variable, and scientific literacy and endorsement of scientific inquiry as predictor variables. PISA datasets contain five sets of plausible values (representing Rasch model estimates) based on the version of the test completed. As recommended, we analysed each value separately and present averaged results [31]. In the final model, students were nested within schools, nested within counties. We allowed slopes and intercepts for the relationship between predictor and outcome variables to vary across each level of data. Due partly to the large dataset, all analyses produced statistically significant results. Thus, we compared the effect sizes for the relationship between predictor and outcome variables, indexed by estimated correlations (*r*), with values of 0.1, 0.3 and 0.5 indicating weak, moderate and strong effects, respectively [32]. The electronic supplementary material contains additional details about these analyses.

As predicted, support for pro-environment policies was more strongly related to endorsement of scientific inquiry than to scientific literacy. A model including both scientific literacy and endorsement of scientific inquiry fit the data better than either scientific literacy, $\Delta \chi_{(3)}^{2}=73\;628,$ *p* < 0.001, or endorsement of scientific inquiry alone, $\Delta \chi_{(3)}^{2}=6674,$ *p* < 0.001. However, whereas support for pro-environment policies was strongly related to endorsement of scientific inquiry, *B* = 0.43, *r* = 0.53, it was only weakly related to science literacy, *B* = 0.12, *r* = 0.16. Thus, for every point endorsement of scientific inquiry rose, support for pro-environment policies rose by 0.43 points on average. Results were relatively consistent across countries. The best fitting model allowed both the intercepts and regression slopes for scientific literacy and endorsement for scientific inquiry to vary across countries and schools. Table 2 shows the standard deviations of the regression lines across countries and schools: the SD_COUNTRY_ scores indicate the standard deviation of the regression slope between countries, and the SD_SCHOOL_ term indicates the standard deviation of the regression slope within schools nested within countries.

**Table 2.** Environmental responsibility scores predicted by scientific literacy and endorsement of scientific inquiry in three-level multilevel models (students nested within schools nested within countries).

	support for pro-environment policy			
model	*B*	SD_COUNTRY_	SD_SCHOOL_	*R*
scientific literacy	0.123	0.063	0.071	0.160
endorsement of scientific inquiry	0.433	0.071	0.077	0.530

Critically, in terms of comparability with the experimental data from Study 2, in the United States support for pro-environmental policies was also strongly predicted by endorsement of scientific inquiry, *B* = 0.52, *r* = 0.60 and only weakly related to science literacy *B* = 0.08, *r* = 0.10.

Several limitations of Study 1 were addressed in Study 2. First, Study 1 was correlational in nature, and therefore does not illuminate whether endorsement for scientific inquiry affects support for pro-environmental policies. Second, Study 1 contained limited demographic controls. Finally, the study investigated the attitudes of adolescents. It would be helpful, from an applied perspective, to understand the relationship in adults (i.e. those most likely to influence policy decisions). To address these issues, we conducted an experiment to test the relationship between endorsement for scientific inquiry and support for pro-environment policy.

## 2. Study 2

Study 1 demonstrated that, compared with scientific literacy, endorsement of scientific inquiry more strongly predicted support for pro-environment policies in a sample of adolescents. In Study 2, we further investigated this idea with an experiment testing whether a simple intervention that enhances endorsement of scientific inquiry would indirectly increase support for pro-environment policies in an adult sample. Participants in Study 2 were presented with either a fact sheet about scientific inquiry (designed to increase endorsement of scientific inquiry) or a fact sheet about scientific research on sleep (control condition). Participants then completed measures of endorsement of scientific inquiry, support for pro-environment policies and scientific literacy, along with measures of egalitarian–communitarian and hierarchical–individualistic world views [16]. Study 2 advances our understanding of the theoretical link between endorsement of scientific inquiry and support for pro-environment policy, and the potential applied value of increasing endorsement of scientific inquiry. Moreover, Study 2 extends our findings to an adult sample, which is critical for building policy support as in most cases adults will be the population most likely to influence policy decisions.

### 2.1. Method

#### 2.1.1. Participants

Using SurveyMonkey audience targeting, we recruited 215 adult US volunteers who were randomly allocated to one of two conditions in an online experiment. Data were only analysed after all 215 participants had been collected.

#### 2.1.2. Materials and procedure

Upon accessing the survey, participants were randomly assigned to read one of two fact sheets. Participants in the experimental condition read the fact sheet about scientific inquiry, and control condition participants read the fact sheet about sleep (described below). Participants in each condition then answered a brief set of questions relating to the fact sheet they had read. All participants then answered the questions probing their endorsement of scientific inquiry, their basic scientific literacy and their support for pro-environment policies. Finally, participants answered the questions indexing the extent to which they adopted a hierarchical–individualist or egalitarian–communitarian world view [16]. Fact sheets, and the associated questions, for both conditions are available in the electronic supplementary material.

##### 2.1.2.1. Scientific literacy scale

We assessed scientific literacy using seven items from the National Science Framework science indicators [7]. These indicators included seven questions, assessing basic understanding of scientific phenomena, which participants judged to be true or false (table 3). Thus, scores for scientific literacy ranged from 0 to 7.

**Table 3.** Items from the scientific literacy, endorsement of scientific inquiry and support for pro-environment policy scales used in Study 2.

scientific literacy items
(a) The centre of the Earth is very hot.
(b) All radioactivity is manmade.
(c) It is the father's gene that decides whether the baby is a boy or girl.
(d) Lasers work by focusing soundwaves.
(e) Electrons are smaller than atoms.
(f) Antibiotics kill viruses as well as bacteria.
(g) The continents on which we live have been moving their locations for millions of years and will continue to move in the future.

endorsement of scientific inquiry items
(a) The development of early warning systems for natural disasters should be based on scientific research.
(b) Complementary and alternative medicines should not be subjected to scientific investigation.
(c) New forms of disease resistant crops do not need to be subjected to scientific research.
(d) Scientific research to develop vaccines for new strains of influenza brings significant public health benefits.
(e) The cause of a disease can only be identified by scientific research.
(f) Statements about the nutritional quality of food should be based on scientific research.
(g) The effectiveness of unconventional treatments for diseases should be subject to scientific investigation.
(h) Science cannot predict complex events like the spread of forest fires.

support for pro-environment policy items
(a) I am in favour of having laws that regulate factory emissions even if this would increase the price of products.
(b) It is not important for governments to invest in renewable sources of energy.
(c) Governments should implement policies that encourage companies to generate electricity from renewable sources.
(d) It is important for electricity to be generated from renewable sources even if it is more expensive to produce.
(e) Regular checks on emissions from cars should be required as a condition of their use.
(f) It is important for governments to implement policies that facilitate the reduction of private citizens’ vehicle emissions
(e.g. by investing in public transport and cycling infrastructure).
(g) Governments should not set greenhouse gas emission reduction targets for industries in order to limit climate change.

##### 2.1.2.2. Endorsement of scientific inquiry scale

We assessed endorsement of scientific inquiry using a novel scale comprising eight items adapted from the PISA items used in Study 1 (table 3). To increase generalizability, we modified items to include domains of scientific research not covered in the original PISA items (e.g. crop production; nutritional content of food). For each item, participants rated their agreement from 1—*strongly disagree* to 7—*strongly agree*. Scores on this scale could thus range from 8 to 56. The endorsement for scientific inquiry scale showed good internal reliability, *α* = 0.79.

##### 2.1.2.3. Support for pro-environment policies scale

We adapted the PISA environmental responsibility items used in Study 1 to provide seven items assessing policy support for environmental policies implicitly—but not explicitly—related to counteracting climate change (table 3). Again, participants rated their agreement from 1—*strongly disagree* to 7—*strongly agree*, producing a score ranging from 7 to 49. Both the endorsement for scientific inquiry and support for pro-environmental policy scales include reverse coded questions. The support for pro-environmental policies scale showed excellent internal reliability, *α* = 0.87.

##### 2.1.2.4. Hierarchicalism and individualism scales

To test whether the effects of our intervention varied depending on participants' world views, we used Kahan *et al*.'s [16] scale to assess the extent to which participants adopted a hierarchical–individualist or egalitarian–communitarian world view. We included six items assessing hierachicalism/egalitarianism (e.g. *our society would be better off if the distribution of wealth was more equal*) and six items assessing individualism/collectivism (e.g. *the government interferes far too much in our everyday lives*). For each item, participants indicated the extent of their agreement on a scale from 1—*strongly disagree* to 6—*strongly agree*. Thus, possible scores on the hierachicalism and individualism scales each ranged from 6 to 36, with higher scores indicating more hierarchical and individualistic world views.

##### 2.1.2.5. Scientific inquiry fact sheet

We developed a fact sheet intended to elucidate the value of scientific inquiry. The fact sheet described (i) different ways of acquiring knowledge (intuition, logic and observation); (ii) scientific research as a way of acquiring knowledge through a combination of logic and observation; (iii) a classic example of scientific research (Semmelweis' work on the benefits of hand-washing); and provided (iv) a brief explanation of the utility of scientific research for acquiring knowledge about some things (whether medical treatments work) but not necessarily others (whether you like a particular piece of art). Following the passage of text, to further engage participants with the information they had read, questions assessed participants' perceptions of the utility of scientific research for acquiring knowledge in four different domains (including determining which of two medications is more effective, and whether you like a particular food).

##### 2.1.2.6. Control condition fact sheet

The control condition fact sheet of approximately equivalent length (Experimental: 574 words; Control: 584 words) provided facts about sleep, and the measurement of sleep using polysomnography. Thus, this fact sheet contained scientific content, but did not address the value of scientific inquiry. This fact sheet was followed by questions assessing participants' perceptions of the utility of various indices for measuring sleep activity.

### 2.2. Results

Consistent with recommendations [33], we adopted conservative criteria for the determination of significant skewness and kurtosis (*p* < 0.001). The data from the endorsement for scientific inquiry and pro-environment policy scales were significantly negatively skewed, *p* < 0.001. We calculated reflected square roots of the scales and then reflected the data again so that higher scores remained indicative of greater endorsement. In both cases, this reduced the negative skew of the data, *p* > 0.001.

**Figure 1.**
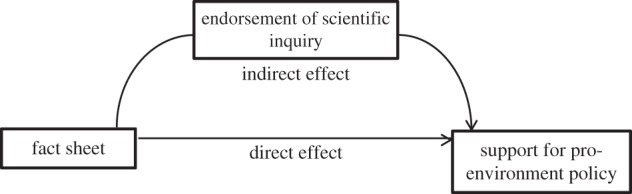
The Mediational Model tested in the present reanalysis. The critical pathway is the indirect effect of the fact sheet on support for pro-environment policy as mediated by participants' endorsement of scientific inquiry.

In accordance with current recommendations of best practice, bootstrapped mediational analyses using 2000 iterations [34] were adopted. Bootstrapped analyses draw *n* (in this case 2000) random samples (termed an iteration) from the data averaging the analysis across each of these samples, thereby increasing the accuracy of the point estimates. These analyses are more appropriate than earlier stepped regression procedures for estimating mediational effects [34]. For a detailed discussion of this method, see Hayes [34]. In our study, the analyses determined the (i) effect of the fact sheet manipulation on endorsement of scientific inquiry; (ii) relationship between endorsement of scientific inquiry and support for pro-environment policy; and (iii) indirect effect of the manipulation on support for pro-environment policy via differences in endorsement of scientific inquiry. Graphically, this analysis is represented by figure 1.

The results supported our hypothesis. The critical predicted indirect effect was clearly present. Reading the scientific inquiry (cf. control) fact sheet increased endorsement of scientific inquiry, *B* = 0.43, 95% CI [0.09, 0.76], *d* = 0.31 [0.15, 0.48], with participants who read the scientific inquiry fact sheet scoring higher on the measure (*M* = 47.31, s.d. = 7.88) than participants in the control (*M* = 44.96, s.d. = 8.05). This, in turn, was associated with greater support for pro-environment policy, *B* = 0.52 [0.38, 0.66], *r* = 0.43 [0.31, 0.53]. The moderately sized indirect effect of the manipulation on support for pro-environment policies via endorsement of scientific inquiry, *B* = 0.22 [0.05, 0.41], *k*^2^ = 0.08 [0.02, 0.15],^1^ did not vary with participants' reported world views (*p*s > 0.10), with participants low, moderate and high in hierarchical individualism demonstrating a similar positive effect of the manipulation on policy support (table 4).

**Table 4.** The indirect effect of the manipulation on support for environmental policies to counteract climate change for participants low, moderate and high in hierarchical individualism.

hierarchical individualism	indirect effect of manipulation on environmental policy support	95% CI of the indirect effect
−1 s.d.	0.18	[0.05, 0.41]
0	0.17	[0.05, 0.35]
+1 s.d.	0.16	[0.05, 0.37]

Additional analysis confirmed that the fact sheet manipulation had virtually no effect on participants' basic science literacy scores, *B* = 0.09 [−0.21, 0.39], *d *= 0.01 [−0.12, 0.14] and that our dataset included a broad range of world views (see the electronic supplementary material for histograms of responses on the hierarchicalism and individualism scales).

Our data showed a clear indirect effect consistent with our hypothesis. This indicates that to the extent to which the manipulation increased endorsement of scientific inquiry, this translated to an increase in support for pro-environment policy. However, another aspect of the results must be considered when evaluating our findings. Specifically, the coefficient for the direct effect (i.e. the effect of the fact sheet on support for pro-environmental policies when the indirect effect is statistically controlled) was *B* = −0.26 [−0.62, 0.09]. The confidence intervals around this coefficient overlap zero, indicating that we cannot assume the effect is meaningfully different from zero. However, the direction of this effect was negative, suggesting that we cannot rule out the possibility that our fact sheet manipulation may have triggered some unanticipated mechanism (other than increased endorsement of scientific inquiry) that suppressed an increase in support for pro-environment policy. Regardless, the indirect effect is the most relevant to our hypothesis and provides clear evidence that, to the extent that the manipulation increased endorsement, this translated to an increase in support for pro-environment policy. Moreover, this result was produced by a manipulation that involved presentation of a brief fact sheet; future research may show that a more comprehensive intervention is more effective.

## 3. General discussion

Whereas previous work has shown that an understanding of basic scientific facts does not necessarily entail greater support for pro-environmental policy [16], we focused on a related concept: endorsement of scientific inquiry. Study 1 demonstrated that across a large, international sample of adolescents, support for pro-environment policy was much more strongly related to endorsement of scientific inquiry than to scientific literacy. Study 2 demonstrated that a simple, low-cost fact sheet on the importance of the scientific method increased endorsement of scientific inquiry and, subsequently, support for pro-environment policies in adults. These studies demonstrate that the results are consistent across adolescent and adult populations. Importantly, the relationship held regardless of participants' endorsement of hierarchical–individualist or egalitarian–collectivist views. From an applied perspective, this is particularly encouraging given recent suggestions that interventions increasing basic science literacy might polarize people's attitudes to climate change according to their world views [16,35].

Consistent with the view that source credibility is important for public acceptance of scientific evidence, increasing endorsement of scientific inquiry as a method of knowledge accumulation also increased acceptance of pro-environmental policies. In terms of theoretical implications, our demonstration that increasing endorsement of scientific inquiry increased support for pro-environment policy—and that these effects were not dependent on concomitant effects on science knowledge—is the first to isolate the contribution of this aspect of science literacy to public decision-making. However, there is an important caveat to this finding. Our manipulation increased endorsement of scientific inquiry but did not increase scientific knowledge. We acknowledge that increasing endorsement of scientific inquiry is unlikely to boost support for pro-environment policy in an audience that is entirely ignorant of what science has concluded about a particular issue (e.g. climate change). For science to effectively inform public decision-making, the public must not only appreciate the scientific methods used in research, but also know something about what this research concludes. Nonetheless, when attempting to garner public support for scientifically motivated policies, assuming the public has some understanding of the relevant scientific consensus, efforts to educate the public regarding the merits of the scientific process might be more fruitful than simply relaying factual information gained from this process.

Our results extend knowledge of the influence of political and cultural factors on support for pro-environment policy. Prior research [16,36,37] suggests that providing information about the risks of climate change can cause public attitudes to climate change to divide along socio-political lines, such that hierarchical individualists (who value industry and commerce) and egalitarian communitarians (who value equality of rights and distribution of resources) become further apart in their attitude towards climate change and policies to mitigate its effects. This is thought to occur because information about climate change has cultural meaning (e.g. it implies that restrictions should be placed on industry, which contradicts hierarchical–individualist values), and people are motivated to interpret information in a way that is consistent with their world view (e.g. hierarchical individualists will judge the risks associated with climate change to be less, because this reduces the need to restrict industry) [36,37].

Crucially, however, it is possible to present information about climate change in a way that counteracts its associated cultural meaning. For example, highlighting geo-engineering approaches to mitigating the effects of climate change (which align with pro-industry values) can make hierarchical individualists more receptive to scientific information about the risks of climate change [37], effectively communicating the scientific consensus on the issue increases climate change belief across socio-political ideologies [38–40] and framing climate change action as patriotic [41,42] or as obeying authority or defending natural purity increases support for such policies [42]. Our results suggest that highlighting the benefits of scientific inquiry may play a similar role. The notion that scientific research is a valid way of accumulating knowledge may appeal equally to values held by hierarchical individualists and egalitarian communitarians. In turn, highlighting the benefits of scientific inquiry may counteract any negative effects of the cultural meaning associated with judgements about climate change risk, and make people who hold either world view more receptive to information about the risks of climate change or more supportive of pro-environment policies. Our findings also suggest interesting possibilities for future research. We deliberately omitted any mention of ‘climate change’ from our experimental materials. Instead, we focused on support for pro-environment policies that were implicitly, but not exclusively, linked to climate change. Thus, we cannot speak directly to the effects of increasing endorsement of scientific inquiry on attitudes towards climate change. However, given our manipulation increased support for these policies, it begs the question: can acceptance of scientific consensus on climate change (and other contentious scientific issues) be increased by educating the public on the benefits of the scientific method?

Deliberately, our manipulation contained little-to-no information indicating a particular socio-political viewpoint, something atypical in real-world settings. A source's perceived political affiliation might interfere with the effects observed in this study [22]. For example, a hierarchical individualist may show less change in endorsement of scientific inquiry and environmental policy support if the message were delivered via a left-wing newspaper. This consideration, though important, was not our focus. Precisely because political source factors can influence the persuasiveness of a message (and the likelihood of further elaboration), we investigated whether simple interventions from *politically neutral* sources could increase support for pro-environment policies via endorsement of scientific inquiry. Our results suggest they can. This underscores the importance of politically independent, moderate institutions in informing the public about the importance of scientific inquiry.

The present findings, while promising, are subject to limitations which are important to note. First, we note that there is scope to further develop the construct of endorsement of scientific inquiry and the scales used to measure it. We have provided a working definition of this construct and a description of how it differs from related but distinct constructs that reflect aspects of scientific literacy and attitudes to scientific research. However, this definition may be refined through future research. Second, it is important to further investigate the effects of interventions designed to increase endorsement of scientific inquiry. Although our study shows that a brief—and therefore cost-effective—intervention can increase endorsement of scientific inquiry and, hence, support for pro-environment policy, it is unclear whether a more comprehensive intervention will produce more substantial changes, whether these are likely to persist over time. Future research should investigate the long-term effects of interventions on endorsement for scientific inquiry and support for pro-environment policy, and also any additional effects that such interventions might have (other than affecting endorsement of scientific inquiry). Finally, in the real world, it is not often the case that information is delivered by sources that are perceived to be politically neutral. One question is whether the effects we demonstrate would occur if delivered by a message source perceived to be politically biased.

Successfully implementing pro-environment policies (e.g. to mitigate the effects of climate change) requires public support [3,4]. Simple interventions increasing endorsement of scientific inquiry may represent cost-effective methods for increasing public support for such policies.

## Supplementary Material

Drummond Palmer and Sauer Addtional Online Supplementary Material.doc - a file containing details of supplementary analyses and materials

## Supplementary Material

Science & Views All Data RSOS.Sav - an SPSS data file of all the data collected for Study 2.

## Footnotes

1 Analysis using the raw scores rather than the square root transformed data were similar, with the indirect effect being slightly larger *k*^2^ = 0.09 [0.02, 0.17].

## Data accessibility

Data for Study 1 are publically available from the Programme of International Student Assessment (http://www.oecd.org/pisa/pisaproducts/). The data from Study 2 are available in the electronic supplementary material. The electronic supplementary material contains additional details of methodology and additional analyses.
