# Supplementary material for: Enhancing endorsement of scientific inquiry increases support for pro-environment policies
Source: R Soc Open Sci. 2016 Sep 28;3(9):160360. doi: 10.1098/rsos.160360 (PMC5043319; doi:10.1098/rsos.160360)
Supplement: Drummond Palmer and Sauer Addtional Online Supplementary Material.doc - a file containing details of supplementary analyses and materials [file rsos160360supp1.docx]

**Study 1 Additional Analyses**

**Science Literacy and Endorsement of Scientific Inquiry**

The correlation between basic science literacy and endorsement of scientific inquiry was moderate, *B* = 0.366, *r* = 0.44. We interpreted effect sizes with 0.1, 0.3 and 0.5 representing small, moderate and large effects respectively [S1]. This represents a conservative criterion (given that multi-level model coefficients could be interpreted as part correlations), but adopting more lenient criteria made negligible difference to results reported in the main text, and only slightly increased the relative effect size of our supplemental analyses. The correlation shows that these two components of science literacy co-vary. However, the correlation coefficients also indicate that the constructs of science literacy and endorsement of scientific inquiry are distinct: reflecting different components of the broader construct of science literacy.

**Support for Climate Change Relevant Policies**

Within the *support for pro-environment policy* scale, three items were of particular relevance to climate change. These related to support for public policy regulating (a) vehicle emissions, (c) factory emissions, and (g) electricity generation from renewable sources irrespective of increased consumer costs. We repeated the primary analyses on the relationship between endorsement for scientific inquiry and pro-environment policy support using only these three items that were directly relevant to the climate change policy. The results from these analyses were qualitatively similar to the analyses considering the complete scale. As in the primary analyses, including both scientific literacy and endorsement of scientific inquiry fit the data better than either scientific literacy (Δχ^2^ (3) = 52,025.38, *p* < .001) or endorsement of scientific inquiry (Δχ^2^ (3) = 4020.99, *p* < .001) alone. Support for policies relevant to climate change was only weakly related to science literacy, *B* = 0.10, *r* = .12, but was moderately related to endorsement of scientific inquiry, *B* = 0.38, *r* = .43.

**Study 2 Fact Sheet Manipulation**

The fact sheets given to participants in the experimental and control conditions were as follows.

**Experimental Fact Sheet**

How do we understand the world around us? One way for us to understand the world is to use intuition and logic. In many cases, using these tools results is a useful method to understand the world – for example, we intuitively understand that gravity pulls us downward, or it is more difficult to see in dark environments. However, often, the tools of logic and intuition are less useful. For instance, for a long time, a large number of people were convinced that the world was flat, or that the Earth was at the centre of the Universe. These ideas have good logical and intuitive appeal – for instance because it may seem that if we were standing on a curved surface we would slide off, or because from our perspective the Sun and Moon appear to come and go around us. Despite their intuitive appeal, these ideas have been proven incorrect through the process known as the scientific method.

Another example of the usefulness of the scientific method was the work of Ignaz Semmelweis, who pioneered the germ theory of infection. Semmelweis worked in a maternity ward and noticed that doctors were not washing their hands after working with corpses. Semmelweis hypothesised that these doctors might be transferring some sort of infection from the corpses to the maternity wing, which had an unfortunately high death rate. After Semmelweis introduced a hand washing procedure for doctors after they had worked with a corpse, the mortality rate in the maternity ward drastically decreased. Thus, Semmelweis was able to save lives. Notably, it was by using the scientific method that Semmelweis was able to detect and counteract an invisible cause of infection. Thus, when intuition fails us, and we are unable to personally see the cause of something, science is a highly important source of information.

The scientific method essentially combines observation and logic in order to understand the world. Scientists use a combination of observation and logic to develop scientific theories. Next, scientists make predictions based on these theories, and test these predictions through further observation. This rigorous process results in scientific theories that describe the world with a high degree of accuracy in comparison to other methods of thinking. The knowledge that science generates also allows scientists to predict, with a high degree of accuracy, the most likely outcome based on a particular set of variables.

In their everyday lives, people make decisions that science may or may not be able to provide useful information about. When we want to make good decisions, the best method of knowing the world will depend upon the specific decision we want to make, and the information available to us when making the decision. For example, if we want to know what potentially harmful side effects a new medication might have, the work of scientists studying the medication would be especially useful. In this case, scientists can apply the scientific method to accurately measure the harmful side effects of the drug through systematic testing, and we can be confident that the measured effects will be consistent across patients. On the other hand, science may be less useful if we are trying to discern whether we personally like a particular work of art. In this case, people’s preferences play a large role in their decisions, and these preferences may vary from person to person. Take a moment to consider in what areas of life the scientific method might be most useful.

Following this paragraph, participants were asked to indicate how useful (on a scale from 1 - *not at all useful* to 5 - *extremely useful*) the scientific method was for four different tasks. These were a) *determining the best design for child safety restraint devices (child seats/capsules) for use in cars*; b) *determining whether you like a particular food*; c) *determining whether you should wear warm or cold weather clothes*; and d) *determining which of two medications is the most effective treatment for a medical condition.*

**Control Fact Sheet (adapted from http://science.howstuffworks.com/life/inside-the-mind/human-brain/sleep.htm)**

Sleep is one of those funny things about being a human being -- you just have to do it. Have you ever wondered why? And what about the crazy dreams, like the one where a bad person is chasing you and you can't run or yell. Does that make any sense?

If you have ever wondered about why people have to sleep or what causes dreams, then read on. In this article, you'll find out all about sleep and what it does for you.

Characteristics of Sleep

We all know how sleep looks -- when we see someone sleeping, we recognize the following characteristics:

• If possible, the person will lie down to go to sleep.

• The person's eyes are closed.

• The person doesn't hear anything unless it is a loud noise.

• The person breathes in a slow, rhythmic pattern.

• The person's muscles are completely relaxed. If sitting up, the person may fall out of his or her chair as sleep deepens.

• During sleep, the person occasionally rolls over or rearranges his or her body. This happens approximately once or twice an hour. This may be the body's way of making sure that no part of the body or skin has its circulation cut off for too long a period of time.

In other words, a sleeping person is unconscious to most things happening in the environment. The biggest difference between someone who is asleep and someone who has fainted or gone into a coma is the fact that a sleeping person can be aroused if the stimulus is strong enough. If you shake the person, yell loudly or flash a bright light, a sleeping person will wake up.

Reptiles, birds and mammals all sleep. That is, they become unconscious to their surroundings for periods of time. Some fish and amphibians reduce their awareness but do not ever become unconscious like the higher vertebrates do. Insects do not appear to sleep, although they may become inactive in daylight or darkness.

By studying brainwaves, it is known that reptiles do not dream. Birds dream a little. Mammals all dream during sleep.

Different animals sleep in different ways. Some animals, like humans, prefer to sleep in one long session. Other animals (dogs, for example) like to sleep in many short bursts. Some sleep at night, while others sleep during the day.

If you attach an electroencephalograph to a person's head, you can record the person's brainwave activity. An awake and relaxed person generates alpha waves, which are consistent oscillations at about 10 cycles per second. An alert person generates beta waves, which are about twice as fast.

During sleep, two slower patterns called theta waves and delta waves take over. Theta waves have oscillations in the range of 3.5 to 7 cycles per second, and delta waves have oscillations of less than 3.5 cycles per second. As a person falls asleep and sleep deepens, the brainwave patterns slow down. The slower the brainwave patterns, the deeper the sleep -- a person deep in delta wave sleep is hardest to wake up.

At several points during the night, something unexpected happens -- rapid eye movement (REM) sleep occurs. Most people experience three to five intervals of REM sleep per night, and brainwaves during this period speed up to awake levels. If you ever watch a person or a dog experiencing REM sleep, you will see their eyes flickering back and forth rapidly. In many dogs and some people, arms, legs and facial muscles will twitch during REM sleep.

Following this paragraph, participants were asked to indicate how useful (on a scale from 1 - *not at all useful* to 5 - *extremely useful*) four different indicators were that a person was asleep. These were a) *the person is lying down*; b) *The person is breathing in a slow rhythmic pattern*; c) *the person’s eyes are closed and their eyes are flicking back and forth beneath their eyelids quickly*; and d) *the person’s brainwaves display delta waves*.

**Hierarchical Individualism**

We measured participants’ Hierarchical Individualism using the scales developed by Kahan et al. (S2). Histograms indicated that we had a wide range of sociopolitical views in our data (see Figures S1 and S2).

*Figure S1.* Histogram depicting percentage of participants with each hierarchicalism score.

*Figure S2.* Histogram depicting percentage of participants with each individualism score.

**References**

1. Tabachnick, BG, & Fidell, LS (2007). *Using multivariate statistics* *5^th^ Edition*. Boston: Pearson.
2. Kahan, DM, Peters, E, Wittlin, M, Slovic, P, Ouellette, LL, Braman, D, Mandel, G. 2012. The polarizing impact of science literacy and numeracy on perceived climate change risks. *Nat. Clim. Change* **2**, 732-735.
